# Genus *Amorphophallus*: A Comprehensive Overview on Phytochemistry, Ethnomedicinal Uses, and Pharmacological Activities

**DOI:** 10.3390/plants12233945

**Published:** 2023-11-23

**Authors:** Fahadul Islam, Rafiuddin Khan Labib, Mehrukh Zehravi, Mashia Subha Lami, Rajib Das, Laliteshwar Pratap Singh, Jithendar Reddy Mandhadi, P. Balan, Jishan Khan, Sharuk L. Khan, Firzan Nainu, Mohamed H. Nafady, Safia Obaidur Rab, Talha Bin Emran, Polrat Wilairatana

**Affiliations:** 1 Department of Pharmacy, Faculty of Allied Health Sciences, Daffodil International University, Dhaka 1207, Bangladesh; fahadulislamdiu@gmail.com (F.I.); talha_bin_emran@brown.edu (T.B.E.); 2 Department of Pharmacy, Faculty of Pharmacy, University of Dhaka, Dhaka 1000, Bangladesh; 3 Department of Clinical Pharmacy, College of Dentistry & Pharmacy, Buraydah Private Colleges, Buraydah 51418, Saudi Arabia; 4 Department of Pharmaceutical Chemistry, Narayan Institute of Pharmacy, Gopal Narayan Singh University, Sasaram 821305, India; 5 Department of Pharmaceutical Chemistry, Faculty of Pharmaceutical Sciences, Assam Down Town University (AdtU), Gandhinagar 781026, India; 6 Department of Pharmaceutical Chemistry, The Erode College of Pharmacy, Erode 638112, India; 7 Department of Pharmacy, International Islamic University Chittagong, Kumira, Chittagong 4318, Bangladesh; 8 Department of Pharmaceutical Chemistry, N.B.S. Institute of Pharmacy, Ausa 413520, India; 9 Department of Pharmacy, Faculty of Pharmacy, Hasanuddin University, Makassar 90245, Indonesia; 10 Faculty of Applied Health Science Technology, Misr University for Science and Technology, Giza 12568, Egypt; 11 Department of Clinical Laboratory Sciences, College of Applied Medical Sciences, King Khalid University, Abha 61421, Saudi Arabia; 12 Department of Pathology and Laboratory Medicine, Warren Alpert Medical School, Brown University, Providence, RI 02912, USA; 13 Legorreta Cancer Center, Brown University, Providence, RI 02912, USA; 14 Department of Clinical Tropical Medicine, Faculty of Tropical Medicine, Mahidol University, Bangkok 10400, Thailand

**Keywords:** *Amorphophallus*, Araceae, traditional medicine, ethnopharmacology, phytochemistry, ethnobotany

## Abstract

The genus *Amorphophallus* belongs to the family Araceae. Plants belonging to this genus are available worldwide and have been used in traditional medicines since ancient times, mainly in Ayurveda and Unani medical practices. *Amorphophallus* species are an abundant source of polyphenolic compounds; these are accountable for their pharmacological properties, such as their analgesic, neuroprotective, hepatoprotective, anti-inflammatory, anticonvulsant, antibacterial, antioxidant, anticancer, antiobesity, and immunomodulatory effects, as well as their ability to prevent gastrointestinal disturbance and reduce blood glucose. Moreover, *Amorphophallus* species contain numerous other classes of chemical compounds, such as alkaloids, steroids, fats and fixed oils, tannins, proteins, and carbohydrates, each of which contributes to the pharmacological effects for the treatment of acute rheumatism, tumors, lung swelling, asthma, vomiting, abdominal pain, and so on. Additionally, *Amorphophallus* species have been employed in numerous herbal formulations and pharmaceutical applications. There has been no extensive review conducted on the *Amorphophallus* genus as of yet, despite the fact that several experimental studies are being published regularly discussing these plants’ pharmacological properties. So, this review discusses in detail the pharmacological properties of *Amorphophallus* species. We also discuss phytochemical constituents in the *Amorphophallus* species and their ethnomedicinal uses and toxicological profiles.

## 1. Introduction

**Figure 1:**
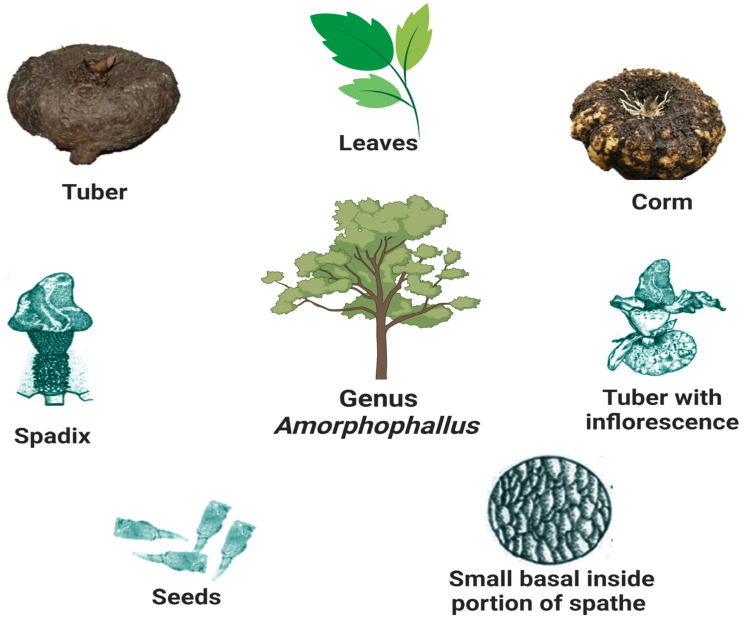
Illustration demonstrates representative icon for Genus *Amorphophallus* and parts of the species as potential sources of bioactive compounds.

*Amorphophallus* is a genus that encompasses 230 species and belongs to the family Araceae. After careful processing to remove harmful ingredients, several species of this genus can be consumed as “famine foods” [1]. *Amorphophallus paeoniifolius* (Dennst.) Nicolson, an aroid more widely known as “Jimikand” or “Elephant foot yam”, is an edible species of *Amorphophallus*. It is one of the less-used aroids. It is a tuberous, stout, native herb commonly used in the Ayurvedic medicinal system to treat numerous human diseases. The demand for this complementary therapeutic has surged [2]. *A. paeoniifolius* is a common tuber crop in sub-tropical and tropical regions worldwide due to its high yield and good taste. It is grown and consumed in significant quantities in countries such as India, Indonesia, Malaysia, and the Philippines. *A. paeoniifolius* has become a cash crop in India because of its high yield, making it popular in the market. As a vegetable, it is prevalent in many Indian dishes. It is rich in protein and starch, and it is also a good source of fiber. It is widely farmed in several Indian states, such as West Bengal, Andhra Pradesh, Uttar Pradesh, Gujarat, Tamil Nadu, Kerala, Maharashtra, and Jharkhand. People living in this area make over INR 100,000 annually [3]. *Amorphophallus* species are also generally available throughout Asia and on Pacific islands. Konjac or *A. konjac* K. Koch is a popular *Amorphophallus* species in China and Japan, with the Chinese having produced this crop for 2000–3000 years. It is also a valuable food crop in Japan, with an extremely high commercial value. Their species names solely distinguish the majority of *Amorphophallus* variants. Only a handful of the 80–90 species are fit for human consumption, with Elephant foot yam and konjac (*A. konjac* K. Koch) being the most popular. Other *Amorphophallus* species have been used ornamentally or for their therapeutic properties. The plants become dormant at the end of the warm, moist growth season, and their corms are harvested [4]. This plant blooms during the rainy season. The flower bud emerges from the corm as a purple shoot, which blooms into a purple flower [5]. The corms (Figure 1) of the *Amorphophallus* species have thermogenic, astringent, anti-inflammatory, anodynic, anti-hemorrhoidal, hemostatic, carminative, expectorant, digestive, stomachic, appetizer, anthelmintic, rejuvenating, aphrodisiac, liver tonic, emmenagogue, and tonic properties. They are beneficial in vitiated Kapha and Vata conditions. They can aid in the treatment of elephantiasis, arthralgia, tumors, inflammations, hemorrhoids, hemorrhages, coughing, vomiting, bronchitis, anorexia, asthma, dyspepsia, colic, flatulence, constipation, splenopathy, seminal weakness, amenorrhea, helminthiasis hepatopathy, fatigue, dysmenorrhea, and anemia [6,7,8].

This review focuses on the ethnopharmacological applications of *Amorphophallus* spp. while also investigating the biological and phytochemical properties of these species. It aims to serve as a foundation for future research on the genus.

## 2. Methodology

To ensure that the most relevant articles were selected for this review, the most common medical, biological, and chemical databases (such as Scopus, PubMed, and Web of Science) were searched using the following primary keywords: “*Amorphophallus*”, “phytochemistry”, and “biological activities”. In addition, “ethnomedical uses” and “safety and toxicity studies” were used as secondary keywords. We selected and assessed review articles and original research articles published in English up to 2023.

## 3. Ethnomedicinal Uses

Historically, the plants of the genus *Amorphophallus* have long been used to treat various diseases. People living in regions such as Bangladesh, India, Nepal, Myanmar, China, Korea, and Indonesia have used these medicinal plants. The *A. konjac*, commonly known as konjac, has been utilized in Japan, China, and Southeast Asia for both dietary and medicinal purposes for an extended period. The flour derived from the corm of this particular species is utilized in Far Eastern cuisine to prepare noodles, tofu, and various types of snacks. For over two thousand years, locals in China have used a flour-based gel to treat asthma, coughing, hernia, breast pain, burns, and hematological and skin disorders. The gel is also employed for detoxification, tumor suppression, blood stasis alleviation, and phlegm liquefaction. Purified konjac flour, known as konjac glucomannan (KGM), has been slowly adopted as a food ingredient and nutritional supplement in the United States and Europe in the last two decades. The latter may be found in capsule form, as well as in beverage mixes and food items. KGM has been shown in clinical research to enhance glucose metabolism, bowel regularity, and colonic ecology, as well as to considerably reduce plasma cholesterol levels [6].

Many reported ethnomedicinal uses of the plants in the genus *Amorphophallus* are highlighted in Table 1.

## 4. Chemical Constituents

### 4.1. Steroids

Steroids are compounds that possess a cyclopentanoperhydrophenanthrene ring. This structure is composed of three 6-carbon rings, denoted A, B, and C, as well as a 5-carbon ring, denoted D. Structurally, steroids are tetracyclic compounds and possess various functional groups that are attached to these rings; these generally include different hydroxyl groups and side chains [15,16].

Phytochemical studies of the genus *Amorphophallus* have revealed that different parts of the plants contain different steroids. *A. commutatus* contains γ-sitosterol (**2**), campesterol (**3**), and stigmasterol (**4**) in its tubers. At the same time, β-sitosterol (**1**) was found in the whole plant and the tubers of *A. campanulatus* [17,18] and *A. paeoniifolius* (Table 2) [7,19], respectively. Steroids are potentially responsible for the analgesic properties of plant extracts, which primarily affect the central nervous system [20]. Steroids from aqueous and methanol extracts of *A. paeoniifolius* tubers may also be responsible for their hepatoprotective effects [21]. Steroids also possess significant antibacterial [22] and anticancer properties [23].

### 4.2. Flavonoids and Other Phenolic Compounds

Flavonoids are compounds found in a variety of vascular plants. Flavonoids possess cytotoxic, anticancer, antioxidant, antibacterial, neuroprotective, and hepatoprotective properties. These effects are mainly attributed to their ability to scavenge free radicals, their tendency to form metal complexes, and their capacity to attach to proteins with high selectivity [42].

Flavonoids possess a flavan nucleus comprising 15 carbon atoms that form a C6–C3–C6 skeleton. The skeleton of a flavonoid is composed of two aromatic rings, denoted as A and B, connected by a 3-length carbon chain. In most flavonoids, the connecting carbon chain bonds with an oxygen atom to create a heterocyclic core or C-ring [43].

**Figure 2:**
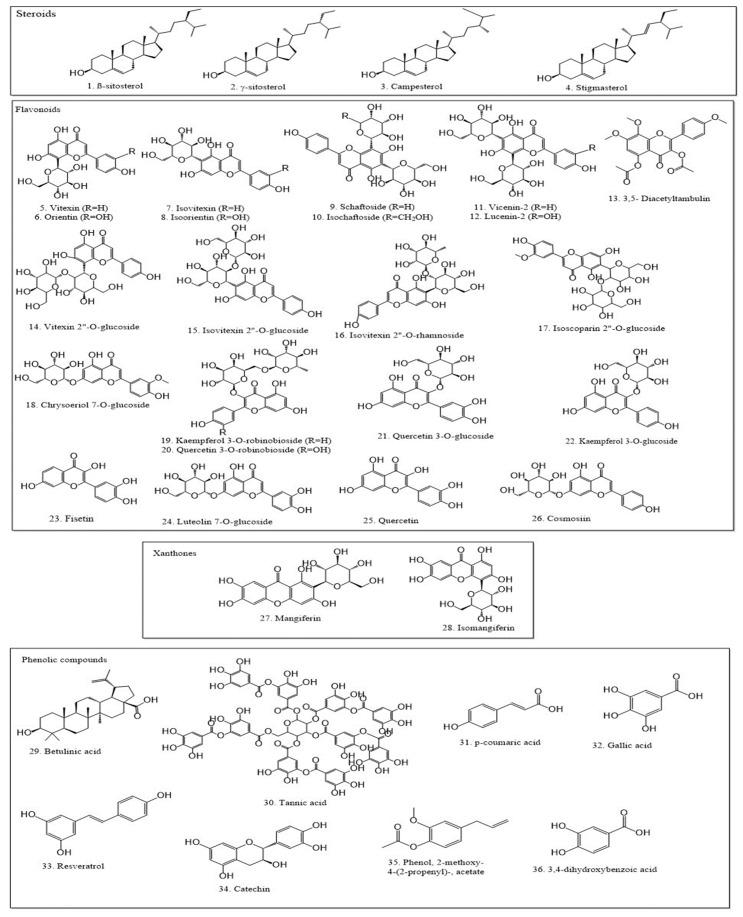

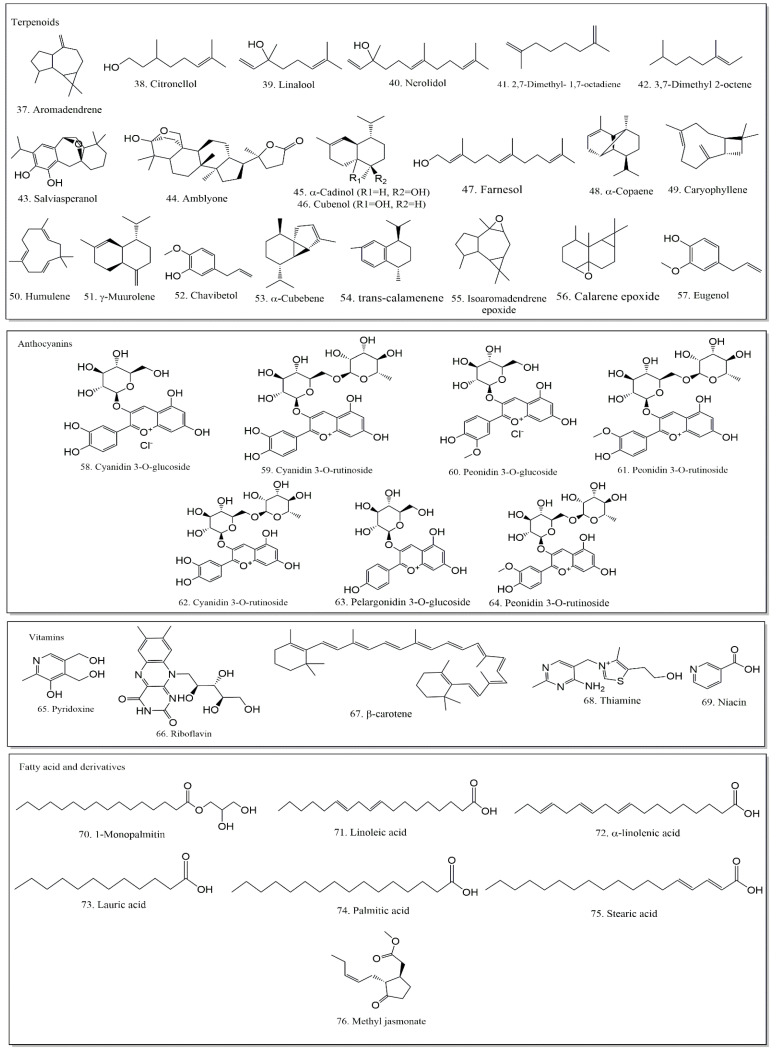

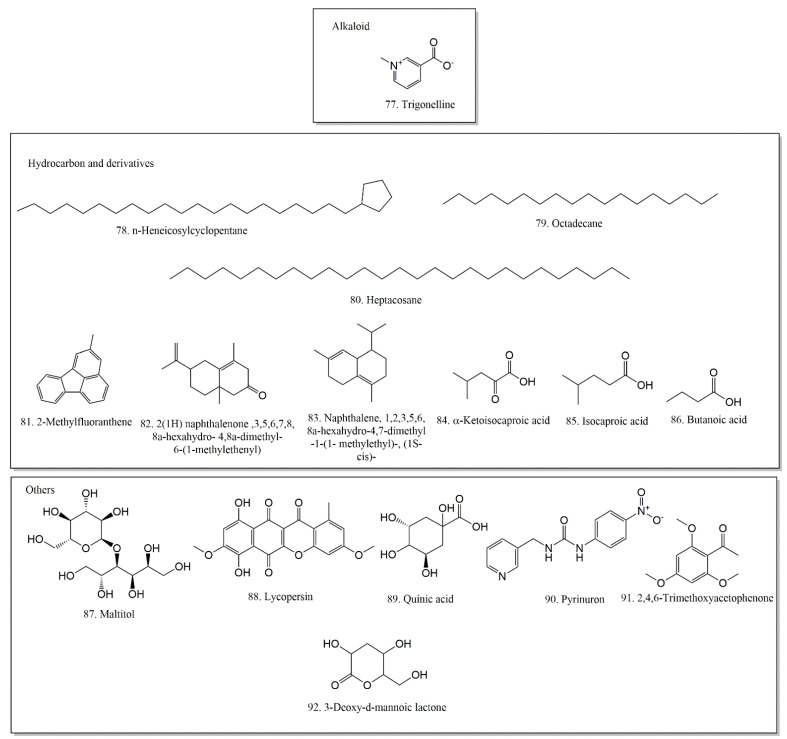
Chemical constituents found in *Amorphophallus* species.

*Amorphophallus* species are a rich source of flavonoids. Several flavonoids (**5**–**26**) (Figure 2), as well as other critical phenolic compounds (**29**–**36**), were isolated from various parts of *A. titanium*, *A. pusillus*, *A. paeoniifolius*, *A. titanium*, *A. rivieri*, and *A. campanulatus* (Table 2) [24,25,26,27,28,31,32,40]. Phenolic compounds were also found in *A. konkanensis* [29], *A. bulbifer* [33], and *A. sylvaticus* [18]. *A. titanium* contained 14 different flavonoid compounds, while *A. pusillus* and *A. paeoniifolius* each contained 13 different flavonoids.

### 4.3. Xanthones

Xanthones possess various pharmacologic effects, including antitumor, antioxidant, anti-allergic, antibacterial, anti-inflammatory, antifungal, and antiviral. They are believed to have the ability to influence cell division, proliferation, apoptosis, inflammation, and metastasis, making them potentially useful as chemopreventive and chemotherapeutic agents [44]. Mangiferin (**27**) and Isomangiferin (**28**) are xanthones that are found in the leaves of *A. titanum* (*Becc.*) and possess the aforementioned biological properties [24].

### 4.4. Terpenoids

Terpenoids make up the largest category of secondary metabolites in plants. They are divided into categories depending on the number of 5-carbon units in their skeletons, including C5, C10, C15, C20, C25, and C30, as well as C40 (carotenoids). Terpene synthase (TPS) is responsible for forming their fundamental backbone structures; these structures are subsequently modified by hydroxylation, dehydrogenation, acylation, and glycosylation. Terpenoids are organic substances that are generally volatile, but non-volatile terpenoids have also been identified (Figure 2) [45].

Many terpenoid compounds were found in the phytochemical analysis of *Amorphophallus* species (**37**–**57**) (Table 2). Terpenoids exhibit antibacterial properties [22].

### 4.5. Anthocyanins

Anthocyanins are natural pigments that are water-soluble and are responsible for the blue, red, and purple coloration of numerous fruits and flowers, which help them attract pollinators [46]. Anthocyanins (**58**–**64**) are also antioxidants that protect plants against oxidative damage [47]. Dietary anthocyanins have been used as protection against some malignancies [48], cardiovascular illnesses [49], and other chronic human issues. These compounds are also known to perform several physiological functions in plants.

Anthocyanins are synthesized by the phenylpropanoid pathway from three molecules of malonyl CoA produced by fatty acid metabolism and one molecule of p-coumaroyl CoA produced from phenylalanine [47]. The anthocyanins found in various *Amorphophallus* species are listed in Table 2.

### 4.6. Vitamins

Vitamins are a group of organic compounds essential to physiological functions in the body. Vitamins are involved in cellular processes and are associated with developing or preventing malignant diseases. Vitamins are essential to the human body, and individuals must meet their daily requirements through dietary sources [50]. Vitamins (**65**–**69**) are found in the tuber of *A. paeoniifolius* (Dennst.) Nicolson, [28] and the stem part of *A. konjac* K. Koch possesses anticancer, immunomodulatory, and anti-inflammatory properties [38,39].

### 4.7. Fatty Acids

Fatty acids serve as both energy sources and components of cellular membranes. These substances possess biological properties that may affect cell and tissue function, metabolism, and responsiveness to hormones and other signals [51]. Fatty acids are present in *Amorphophallus* species. Compounds (**70**–**72**) and (**76**) are found in *A. paeoniifolius* [28,40]. Compounds (**73**–**75**) are present in *A. commutatus* var. wayanadensis [14]. 

### 4.8. Alkaloids

Alkaloids are significant chemical substances that act as a valuable source for the exploration of new drugs. Various alkaloids derived from plant sources demonstrate inhibitory effects on cell growth, bacteria, viruses, insects, and the growth of cancer cells, both in vitro and in vivo [52]. Trigonelline (**77**), an alkaloidal compound, is present in the corm, the dormant stem base part of *A. konjac* K. Koch, and possesses the aforementioned biological properties [38,39].

### 4.9. Hydrocarbon and Derivatives

Hydrocarbons are organic compounds composed of hydrogen and carbon atoms. They are the building blocks of many other organic molecules, and they are found in a wide variety of natural and synthetic materials, including petroleum, natural gas, coal, plastics, and pharmaceuticals [53]. Hydrocarbon and derivatives are found in the tuber of *A. lanceolatus* Hett. and Seeds of *A. sylvaticus* (Roxb.) Kunth. Compounds (**78**–**82**) are found in the tuber of *A. lanceolatus* Hett., and compound (**83**) is present in the seeds of *A. sylvaticus* (Roxb.) Kunth [18,41]. The remaining compounds belong to hydrocarbons (**84**–**86**) and are found in *A. myosuroides***,** *A. angustispathus***,** *A. atroviridis***,** *A. linearis***,** *A. saraburiensis*, and *A. taurostigma* Ittenb [35].

### 4.10. Others

In the case of other chemical components, there are also several categories. For example, compounds (**87**–**90**) are found in the tuber segment of *A. lanceolatus* Hett. They are Maltitol (sugar alcohol), Lycopersin (polyketide), Quinic acid (cyclic polyol), and Pyrinuron (nitroaromatic compound) (Table 2). Compounds (**91** and **92**) are present in the seed part of *A. sylvaticus* (Roxb.) Kunth and spathe of *A. paeoniifolius* (Dennst.) Nicolson, respectively [18,40].

## 5. Pharmacological Properties

### 5.1. Analgesic Activity

Analgesics are medications that are used to alleviate pain. They are associated with several pathologic conditions and diseases. It is essential to identify the conditions in which analgesics are required, such as muscle aches and headaches, while also ensuring that there is no risk of addiction [11]. The analgesic effectiveness of chemical compounds on mice is usually tested using the tail-flick assay and the writhing test, which involves acetic acid. The intraperitoneal administration of *A. paeoniifolius* extracts at 250 and 500 mg/kg body weight (b.w.), and male Swiss albino mice demonstrated that the extracts exhibited significant analgesic properties. Substances that exhibit analgesic characteristics include flavonoids, alkaloids, and steroids. These substances either inhibit the cyclooxygenase enzyme or operate on the µ receptors in the brain [20]. When administered to Swiss albino mice at doses between 200 and 400 mg/kg, *A. commutatus* var. *wayanadensisa* exhibited anti-inflammatory properties by reducing the activity of the TNF-α and COX-2 enzymes [54]. Analgesic effectiveness has also been demonstrated in animal models using methanol extracts of the *A. campanulatus* tuber (ACME) at various dose ranges (50–500 mg/kg) [11].

### 5.2. Neuroprotective Effects

Neuroprotection is defined as the capability of a treatment procedure to stop neuronal cell death by interfering with or inhibiting the pathogenetic cascade that leads to cell dysfunction and potentially eventual death [55]. Petroleum ether extracts (PEEs) of the *A. paeoniifolius* tuber substantially decreased locomotor activity and caused drowsiness at doses between 100 and 1000 mg/kg. This CNS depression could be caused by interfering with the functions of the cortex [56]. By interfering with the opening of the GABA-mediated Cl^−^ channel, the components of the PEEs may hyperpolarize the cells and produce the observed CNS depressive effects. These findings indicate that the PEEs have a depressive effect on the CNS via the GABAA receptor in the same animal models [57]. Additional anxiolytic properties inherent within the PEEs of *A. paeoniifolius*, which contain lipids, steroids, and fixed oils, were demonstrated dose-dependently (100, 150, and 200 mg/kg) [58]. Chen [59] proved that the administration of 100, 200, and 500 mg/kg doses of *A. campanulatus* PEE over 14 days resulted in an observable neuroprotective effect via the lowering of amyloid peptides, oxidative stress (OS), and acetylcholinesterase levels in brain tissue.

### 5.3. Hepatoprotective Activity

Hepatoprotection is the ability of a chemical substance to protect the liver by returning the functions of glutathione peroxidase (GPx), catalase (CAT), and superoxide dismutase (SOD) to normal levels [60]. The methanolic extract of *A. campanulatus* (MEAC) tubers at doses of 250–500 mg/kg in albino Wistar rats shows the hepatoprotective properties of the plant, which caused the levels of SOD, CAT, and GPx in the animal to increase due to the reduction of biochemical markers of hepatic injury, such as serum oxaloacetate transaminase (SGOT), serum glutamate pyruvate transaminase (SGPT), bilirubin (BRN), alkaline phosphatase (ALP), and total protein [61]. In addition, the *A. campanulatus* extracts administered at a dose of 250–500 mg/kg protected hepatic rat tissue against ethanol-induced oxidative damage, most likely by acting as an antioxidant [21]. Flavonoids and steroids from *A. paeoniifolius* tuber extracts may be responsible for their hepatoprotective effect. A 300 mg/kg p.o., the dose of *A. paeoniifolius* given to male albino Wistar rats, exhibited a hepatoprotective effect against paracetamol-induced liver injury [21]. Jain et al. [62] demonstrated that 500 mg/kg orally administered doses of *A. campanulatus* extracts given to male albino Wistar rats exhibited a strong hepatoprotective effect towards CCl_4_-induced hepatic damage because of the presence of flavonoids in the extracts, which can scavenge free radicals. The same ethanolic extract at doses of 250–500 mg/kg also inhibited ethanol-induced oxidative damage in the hepatic tissue of Wistar rats, most likely through antioxidant activity. A possible mechanism includes reducing oxidative damage to hepatocytes by reactive oxygen species (ROS) scavenging activity. It also restores and preserves SOD and CAT activity in liver tissue, normalizes liver marker enzyme levels, and reduces GSH depletion and TBARS formation [63]. The polyphenolic compounds identified in *A. commutatus* extracts were found to exhibit hepatoprotective solid effects; the activities of antioxidant enzymes were elevated, lipid peroxidation was suppressed, and hepatic marker levels decreased in Swiss albino mice models when administered at a dose of 400 mg/kg [64].

### 5.4. Anti-Inflammatory Activity

**Figure 3:**
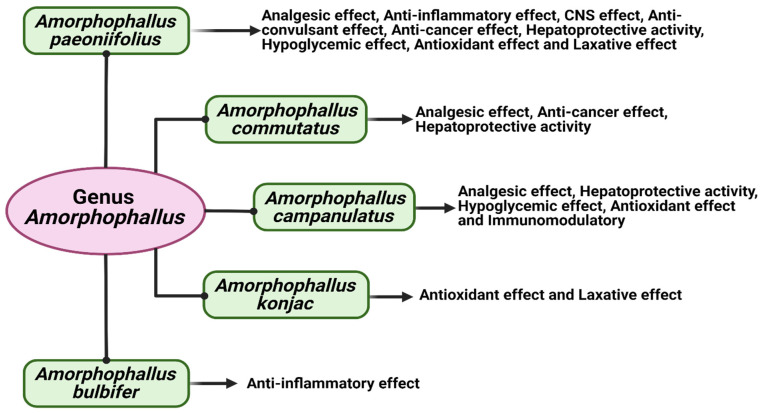
Illustration representing the summary of pharmacological actions of different species of *Amorphophallus* genus.

Any substance that reduces inflammation (pain and swelling) in the body is an anti-inflammatory agent. These treat various disorders by inhibiting the production of certain chemicals that cause inflammation [65]. Inflammation can be reduced by reducing OS [66,67,68]; chronic stresses increase inflammation and thus elevate cortisol levels in the blood [69]. The methanol extract of *A. paeoniifolius* exhibits significant anti-inflammatory activities, while these properties are less significant in the chloroform extract. After three hours of carrageenan administration, methanol extracts were shown to block 37.5% and 45.83% of the carrageenan-induced enzymes compared to controls at 200 and 400 mg/kg, respectively. This is because *A. paeoniifolius* contains alkaloids and flavonoids that exhibit anti-histaminic activities, which inhibit the release of histamine or serotonins [70]. A 200 mg/kg hydroalcoholic extract of *A. bulbifer* also exhibited anti-inflammatory properties by suppressing the prostaglandin synthesis reaction in Wistar rats [13]. In vivo studies using male NC/Nga mice as well as a murine model of human Alzheimer’s disease revealed that skin inflammation and hyper-IgE production were both suppressed when 30 µm, 100 µm, and 160 µm of 5% (*w*/*w*) konjac GM powder was administered. To cure atopic disorders, such as atopic dermatitis, asthma, and allergic rhinitis, the production of IFN-α in the body must be decreased as it is a positive regulatory cytokine of atopic skin inflammation (Figure 3) [71].

### 5.5. Anticonvulsant Effects

The anticonvulsant properties exhibited by PEEs of *A. paeoniifolius* may be due to steroidal compounds. These compounds could aid allosteric receptor facilitation or lessen their inactivation by increasing the synthesis and release of GABA. The PEEs could have a natural effect on the GABAergic system and have been demonstrated to exhibit dose-dependent action regarding convulsion onset in Isoniazid-induced male albino mice models at doses of 200, 300, and 400 mg/kg [72].

### 5.6. Antibacterial Activity

*Escherichia coli, Staphylococcus aureus, Pseudomonas aeruginosa,* and *Streptococcus mutans* are four types of bacteria that were inhibited by 20 μL and 25 μL administrations of ethanolic tuber extracts from *A. paeoniifolius* in an in vitro study using nutrient agar (HiMedia) [73]. Amblyone, a compound separated from *A. campanulatus*, exhibited antibacterial properties against four Gram-positive and six Gram-negative bacteria. Amblyone was most active against *Bacillus megaterium* and least active against *P. aeruginosa*. The substance exhibited excellent antimicrobial properties against the tested microorganisms, moderated cytotoxicity against brine shrimp nauplii, and did not display any antifungal activity against the fungi tested [37]. An amount of 20 µL of a 200 µg/mL solution of *A. paeoniifolius* extract dissolved in DMSO inhibited the bacterial growth of *Bacillus subtillis, K. pneumoniae* MTCC 109, *P. aeruginosa* MTCC 424, and *S. aureus* MTCC 727; this may be due to the presence of phenols, glycosides, polysterols, tannins, flavonoids, terpenoids, gum, steroids, and mucilage in the extract. There was a 7.5 mm zone of inhibition against *B. subtilis* MTCC 121, while *S. aureus* MTCC 737 exhibited the most activity with an ethyl acetate extract (7 mm zone of inhibition). The aqueous extract inhibited *K. pneumoniae* MTCC 109 in a 2.75 mm zone [22]. 25 µL of tuber-mediated nanoparticles synthesized from the tuber extracts of *A. paeoniifolius* exhibited significant antibacterial activity against *B. subtillis, P. aeruginosa, S. aureus, E. coli, S. typhimurium,* and *C. freundii* [74].

### 5.7. Antioxidant Activity

Antioxidants protect cells from free radicals, which are unstable chemicals created by the body in response to environmental stimuli [75,76]. A 1–50 µg/mL ethanol extract from *A. paeoniifolius* administered to Wistar mice significantly inhibited lipid peroxidation by reducing free radical generation by oxidation and exhibiting H_2_O_2_ scavenging activity. Gallic acid, resveratrol, and quercetin were among the phenolic components in the extract responsible for the scavenging activity [33]. A 125–250 mg/kg dose of ACME exhibited hepatoprotective and antioxidant activity by reducing the OS induced by thioacetamide (TAA) in Wistar rats while also reducing the serum ALT, AST, LDH, ALP, and tissue malondialdehyde levels. The levels of glutathione reductase (GR), glutathione (GSH), glutathione-S-transferase (GST), and GPx in the liver and kidneys were also significantly elevated [77]. Similarly, the hexane and methanolic extracts of the *A. campanulatus* tubers at 125 and 250 mg/kg doses exhibited hepatoprotective properties against TAA-induced OS in rats. The methanol extracts radical scavenging and antioxidant activities because of its higher flavonoid and phenolic content than the hexane extracts [78]. The ethanolic extracts of *A. campanulatus* also exhibited antioxidative and antiapoptotic properties in a study involving male albino Wistar rats. Serum urea, tissue TBARS, pro-inflammatory cytokines, creatinine, and GSH metabolizing enzyme activity were dramatically increased, but mitochondrial and cytosolic SOD, CAT, and GSH levels were significantly lowered; hence, this extract may be effective in reducing ACE-induced nephrotoxicity [79].

### 5.8. Prevention of Gastrointestinal Disturbance

Acute diarrhea is a prominent cause of neonatal death, particularly in underdeveloped nations. Traditional practitioners have used various medicinal plants with anti-diarrheal qualities; however, the effectiveness of these anti-diarrheal therapeutic approaches has not been scientifically proven [80]. The methanolic (APME) and aqueous (APAE) extracts of *A. paeoniifolius* exhibited a spasmogenic and stimulatory effect on prostaglandin synthesis due to betulinic acid and glucomannan. These extracts were administered to Wistar rats in 250 and 500 mg/kg doses while inducing gastrokinetic activity, and moderately increased the number of stools, the volume of feces, the water content of the fecal matter, gastric emptying, and intestinal transportation in rats [31]. APMEs at 250 and 500 mg/kg doses reduced free acidity, gastric volume, total acidity, ulcer score, and pH of male albino rats. The protection percentage for 250 mg/kg and 500 mg/kg was 67% and 85.5%, respectively; this may be due to the presence of polyphenols in the extract, which exhibit anti-ulcer properties by increasing GSH levels and inhibiting lipid peroxidation [81]. In conjunction with castor oil, the leaf extracts of *A. paeoniifolius* leaves cause a substantial reduction in the frequency and severity of diarrhea at 100, 200, and 400 mg/kg doses in Swiss albino mice models. Constipated rats treated with 125, 250, and 500 mg/kg of APME and APAE from the plant’s tubers containing glucomannan and betulinic acid significantly improved their fecal parameters and intestinal transit time. Betulinic acid may be responsible for this behavior, as it exhibits a spasmogenic effect induced by partial agonistic action in the 5HT receptors [7]. *A. konjac* flour at 300 and 600 mg/kg b.w. doses proved effective at increasing the water content, fecal pellet weight, and intestinal motility because of its ability to absorb and retain water in the gastrointestinal tract, demonstrating its laxative properties [82].

### 5.9. Anticancer Effect

Cancer is a disease in which a cluster of cells exhibits uncontrolled proliferation (division beyond their normal limits), invasion, and, in certain circumstances, metastasis (the spread of cells beyond their original sites of origin). Cancers are differentiated from benign tumors by their malignant characteristics; unlike cancers, tumors are self-limited, do not penetrate or spread, and have no malignant characteristics [80,83]. Cancer is a multistage process induced by genetic alterations that activate several specific signaling pathways that eventually transform a healthy cell into a cancer cell. Polyphenols and phytosterols, combined with anticancer drugs, can influence numerous cancer-related signaling transduction pathways, which may positively affect cancer treatment [23]. 

#### 5.9.1. Breast Cancer

In a study on estrogen-positive MCF-7 and triple-negative breast cancer, about 10–20 mg/mL of *A. paeoniifolius* (Dennst.) extract was administered to the breast cancer cell line MDA-MB-231 for adhesion, migration, and invasion assays, while 0–500 mg/mL of APTE was administered for a cytotoxic assay. Apoptosis was increased in these cells as evidenced by a decrease in antiapoptotic Bcl-2 and an increase in pro-apoptotic Bax, Caspase-7 expression, PARP breakdown, and decreased motility in both cell lines. Hence, the significant cytotoxic activity exhibited by these extracts was dose- and time-dependent [28]. 

#### 5.9.2. Colon Cancer

In an in vitro study, a 50 μg/mL concentration of the methanolic extract of *A. commutatus* var. *wayanadensis* (ACW) was administered to the HT-29 cell line. MEAC exhibited antioxidant and cytotoxic properties by initiating the caspase-3-dependent apoptotic pathway, inducing a G1/S cell cycle arrest, and subsequently downregulating the COX-2 pathway [84]. A 400 mg/kg dose of the same extract exhibited anticancer properties in tumor-bearing mice models; this was expressed as an increase in antioxidant enzymes, which returned the subject’s hematological profile to normal. The anticancer properties of β-sitosterol extracted from ACW are expressed by the mediation of COX-2 and caspase-dependent pathways [84]. In another in vitro study, the human colon carcinoma cell line HCT-15 was injected with *A. campanulatus* tuber extracts dissolved in 50 μL DMSO. ACME subfractions inhibited the proliferation of HCT-15 cells. They caused dose-dependent apoptosis via the following two fundamental mechanisms: (1) a change in the redox state and (2) interference with essential cellular functions (such as the cell cycle, apoptosis, inflammation, angiogenesis, invasion, and metastasis) [85]. A dose of 250 mg/kg of ACME administered to male Wistar rats also resulted in significant lipid peroxidation (MDA) in the intestine and colon; a reduction in the levels of antioxidants such as CAT, GR, GPx, GSH, and GST; and reduced antioxidant enzyme activity. Thus, this extract has a significant chemopreventive impact on DMH (1,2-dimethylhydrazine)-induced colon cancer [86].

#### 5.9.3. Gastric Cancer

*Amorphophallus konjac* (TuAK) has traditionally been used as an antitumor agent in Chinese medicine. The current investigation examined the inhibitory properties of TuAK against gastric cancer and the associated mechanisms pertaining to two distinct pathways of programmed cell death: apoptosis and autophagy. The abbreviated form of TuAK, known as TuAKe, exhibited a noteworthy suppressive effect on the proliferation of AGS and SGC-7901 gastric cancer cell lines in vitro, with an IC_50_ range of 35–45 µg/mL. The administration of TuAKe can potentially enhance cellular apoptosis and trigger the arrest of the cell cycle. The administration of TuAKe reduced the expression of survivin and Bcl-2, both of which are proteins associated with apoptosis. Conversely, the expression of Bax and caspase-9, which are also apoptosis-associated proteins, increased. Additionally, TuAKe may stimulate autophagy, as evidenced by the substantial reduction in the antitumor effectiveness of TuAKe when autophagy was explicitly suppressed. This implies that autophagy may play a role in the cell death induced by TuAKe. Additionally, individuals diagnosed with gastric cancer who were administered TuAK-based medicinal decoction exhibited enhanced life quality assessment scores in comparison to those who did not receive TuAK treatment. The study exhibited the antineoplastic efficacy of TuAKe against gastric carcinoma and represents the primary documentation to indicate that the fundamental mechanism is linked to the stimulation of autophagy. The data presented provide evidence for the clinical application of medication based on amorphophallus konjac together with conventional chemotherapy to attain an optimized outcome for patients with gastric cancer [87]. 

#### 5.9.4. Hepatocellular Carcinoma

ACMEs that were given orally to male albino Wistar rats at doses of 125 and 250 mg/kg b.w. significantly decreased the NDEA-induced rise in hepatic nodule incidence, nodule multiplicity, and serum biochemical markers, while also significantly enhancing the hepatocellular architecture in a dose-dependent manner. This was the case when the rats were treated with ACMEs. This was made possible by the restoration of antioxidant enzymes such as SGPT, SGOT, ALP, and BRN, in addition to the total proteins, and by the extract’s higher capacity to scavenge peroxide radicals [88]. A study investigated the potential antitumor effects of the organic extract of *A. konjac* tuber (AKT) in two hepatocellular carcinoma cell lines, Huh7 and H22. The H22 cell line was also utilized to establish a mouse tumor model for further evaluation. The apoptotic rate of cells was assessed through the utilization of flow cytometry.

The present study employed immunohistochemistry to investigate the in vivo expression of survivin and Bax proteins associated with apoptosis. AKT demonstrated anti-neoplastic effects against cell lines of hepatocellular carcinoma. The in vivo inhibition of H22 was equivalent to that of 5-fluorouracil. The induction of apoptosis by AKT is attributed to the up-regulation of Bax and down-regulation of survivin. The findings of the study indicate that AKT has a suppressive effect on hepatocellular carcinoma. This effect is attributed to the regulation of survivin and Bax expression. These results lend support to the potential clinical application of AKT-based medication to optimize outcomes in individuals with hepatic carcinoma [89]. Further, in research, the cytotoxic and apoptosis-inducing effects of subfractions of *A. campanulatus* tuber methanolic extract (ACME) were tested in a human liver cancer cell line called PLC/PRF/5. The results showed that these effects were dose-dependent. The findings support the hypothesis that the sub-fractions of ACME inhibit cell development by stimulating the process of apoptosis. The chloroform fraction of ACME was the only sub-fraction that demonstrated a dose-dependent pattern of considerable cell growth inhibition when applied to PLC/PRF/5 cells. However, more investigation is necessary to fully understand the distinct mechanisms through which the sub-fractions of the methanolic extract derived from the tuber of *A. campanulatus* induce cytotoxic and apoptotic effects on the hepatic cancer cell line PLC/PRF/5 [90].

### 5.10. Reduction of Blood Glucose

Glucomannans isolated from the tubers of *A. konjac* have been shown to reduce body weight, minimize the ingestion of meals that increase cholesterol and glucose concentrations, lower the post-prandial rise in plasma glucose and hepatic cholesterol production, and increase the fecal clearance of cholesterol-containing bile acids [91,92]. *A. konjac* extract exhibits hypoglycemic and antioxidant properties and improves glucose metabolism in Wistar rats at doses of 102 mg/kg by suppressing lipid peroxidation as well as inhibiting α-amylase and α-glucosidase activities by 14% and 90%, respectively. This process also includes improving GPx and CAT activities, the reduction of malondialdehyde and lactate dehydrogenase, and aggregating ROS [93]. Due to the inhibitory action of betulinic acid, lupeol, stigmasterol, and β-sitosterol on α-glucosidase and α-amylase, the methanol extract of corms of *A. campanulatus* significantly lowered blood glucose levels in Swiss albino mice models in a dose-dependent fashion [94]. Similarly, glucomannan from a diet of *A. variabilis* may lower blood cholesterol levels, as observed in Wistar rats administered with 60 mg/kg b.w. per day [95]. A combination of mungbean, *Vigna radiata* (L.) *R. Wilczek,* and yam, *A. paeoniifolius* (Dennst.), dramatically lowered cholesterol, triglyceride, and low-density lipoprotein levels while increasing high-density lipoproteins (HDL). There was also a statistically significant (*p* < 0.01) improvement in atherogenic indices when 0.054 g and 0.216 g per 100 g b.w. of this mixture was administered to albino mice models, which was caused by lowering the hormone leptin levels. The hypolipidemic activity of *A. paeoniifolius* glucomannan was comparable to that of statins, involving the competitive inhibition of HMG-CoA reductase and reduced LDL production [96]. In another study, Safitri et al. [97] demonstrated that *Porang* glucomannan administered at doses between 25 and 100 mg/200 g b.w. in the lipid profile of 30 metabolic syndrome-induced Sprague Dawley rats was enhanced by lowering total cholesterol (TC), triglycerides (TG), and LDL, while increasing HDL. Similarly,* A. muelleri Blume* glucomannan fibers were administered in daily doses of 0.06 g/kg b.w. and 0.12 g/kg b.w., which exhibited antidiabetic properties in mice models through the insulin receptor tyrosine kinase pathway. Glucomannan fibers have been shown to boost the expression of IRS-1 and PI3-K, suggesting that they may prevent insulin resistance in diabetic rats [97].

### 5.11. Antiobesity Effect

**Figure 4:**
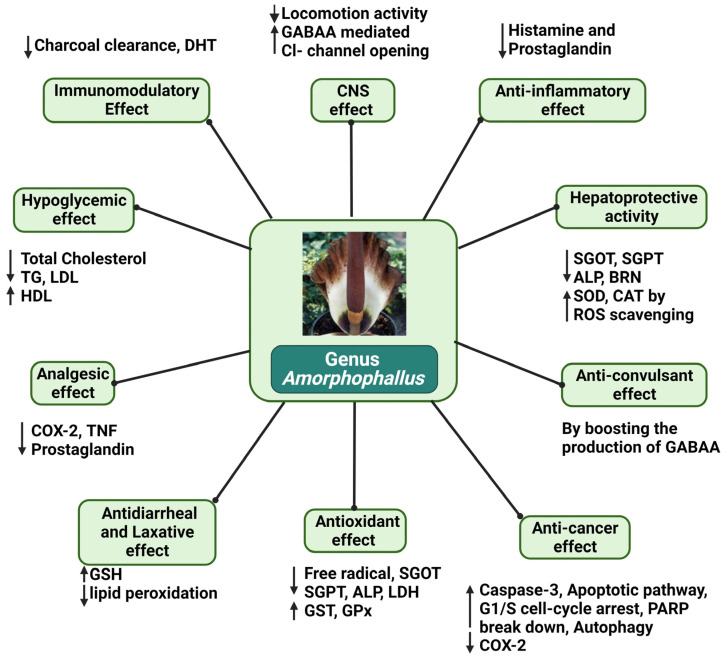
Illustration representing the pharmacological actions of genus *Amorphophallus*. Alkaline phosphatase = ALP, Bilirubin = BRN, Catalase = CAT, cyclooxygenase-2 = COX-2, dihydrotestosterone = DHT, Glutathione peroxidase = GPx, Glutathione = GSH, Glutathione-S-transferase = GST, Glutathione reductase = GR, Low-density lipoprotein = LDL, High-density lipoprotein = HDL, Oxidative stresses = OS, Serum oxaloacetate transaminase = SGOT, Superoxide dismutase = SOD, Serum glutamate pyruvate transaminase = SGPT, Thioacetamide = TAA, Tumor necrosis factor = TNF.

Antiobesity agents are substances that aid in weight loss or maintenance. These medications affect weight regulation by controlling the person’s appetite or calorie absorption, increasing energy consumption, or blocking digestive enzymes [98,99]. When foodstuffs derived from *A. konjac* were mixed with flavonoids and administered to Wistar rats, its antioxidation effects were considerably enhanced, and the serum lipid levels of the rats were reduced; however, the antiobesity effect of the mixture was limited. Specifically, refined *A. konjac* powder decreased MDA levels while significantly elevating SOD and GSH-Px activities. It also lowered LDL, TC, and TG levels while increasing HDL levels, as well as reducing MDA levels while significantly increasing SOD and GSH-Px activities [100]. Extremely obese Sprague Dawley rats that were given 100 g of konjac flour, a powder from the tuber of the *A. konjac*, exhibited lower body weights and total fat wet weight, as well as lowered triglyceride, glucose, and HDL levels in their blood. This was due to a lowered rate of stomach emptying, the binding and removal of bile acids in the gut by transporting them out of the body via feces, and the conversion of more cholesterol into bile acids [38]. Li et al. [101] found that *A. konjac* substantially reduced the body weight of rats and their blood’s triglyceride, glucose, cholesterol, and HDL levels. An amount of 0.2–0.8 g/kg of *A. konjac* administered to 96 Sprague Dawley white rats caused a delay in stomach emptying and more gradual dietary sugar absorption. Keithley and Swanson [102] suggested that 2–4 g per day of glucomannan, combined with a regular caloric or hypocaloric diet, can help people lose weight. The increased viscosity of gastrointestinal content, delayed stomach emptying, decreasing colon transit time, and a reduced rate of meal absorption in the small intestine can lead to attenuated postprandial glucose and insulin surges in individuals who are overweight or obese. An amount of 1.5 g of *A. konjac*, an abundance of glucomannan fibers, was administered daily over 12 weeks to 58 obese subjects; its antihyperlipidemic and antiobesity effects were observed. The cholesterol-lowering action of *Garcinia cambogia* and A.* konjac* combined with their apparent low toxicity suggests that the combined use of substances may significantly benefit the treatment of obesity-related dyslipidemia by promoting weight loss, potentially reducing fatty acid biosynthesis, increasing water absorption, and establishing highly viscous solutions. Its action may have been more effective due to higher stomach volumes and a decreased rate of stomach emptying, which would also alter the kinetics of duodenal fat absorption (Figure 4) [103].

### 5.12. Other Activities

The immunomodulatory efficacy of MEAC from tubers was demonstrated by a substantial reduction in delayed-type hypersensitivity (DTH) response, spleen index, and charcoal clearance in rats administered at doses of 250 and 500 mg/kg, suggesting that the extracts suppressed the immune system in a dose-dependent manner (Figure 4) [104]. *A. paeoniifolius* extracts were administered to *Pheretima posthuma*, the adult Indian earthworm, at doses of 25, 50, and 100 mg/mL, and exhibited anthelmintic efficacy against *Tubifex tubifex* and *Pheretima posthuma*. It not only paralyzed (vermifugal) but also killed the earthworms (vermicidal) (Table 3) [105]. *A. konjac* extract capsules (containing 5 mg glycosylceramides) were given at doses of 100 mg/day to 51 healthy humans and compared to a placebo group; it was observed to improve skin metrics such as dryness, hyperpigmentation, irritation, and oiliness. This may be due to sphingolipids consumed and transformed into sphingolipid metabolites, which are absorbed by the colon and circulated in the blood [106].

## 6. Safety and Toxicity Studies

*A. konjac* has historically been used to make food and medicine, especially in China, Japan, and Southeast Asia [6]. The methanolic (APME) and aqueous (APAE) extracts of *A. paeoniifolius* are safe at doses of up to 2000 mg/kg b.w. (limit test). The LD50 was greater than 2500 mg/kg in Wistar rats [31]. In a Swiss albino mouse model, PEAPs did not demonstrate any toxic effects up to 1.5 g/kg [58]. A research group investigated the acute oral toxicity of *A. oncophyllus* (porang) macerated with *S. crispus* in Wistar rats. The study specifically looked at the death rate, modifications in behavior, kidney and liver function, and urine protein levels. The indicators used were the mortality rate and levels of SGPT and SGOT. The acute toxicity investigation demonstrated that porang and porang macerated with *S. crispus* exhibited no toxicity, even at the maximum dosage of 5000 mg/kg BW. The lack of LD50, unchanged behavior, no weight reductions, and expected findings of biochemical testing, including urine protein, SGPT, and SGOT, provided evidence for this conclusion (Table 4) [107]. Another research assessed the safety of ingesting porang flour with reduced levels of calcium oxalate in Wistar-strain white rats. The experimental design consisted of administering porang flour in five treatments ranging from 0 to 15,000 mg/kg b.w. Each treatment was repeated six times over 60 days. The results indicated no statistically significant difference in body weight, water consumption, calcium, potassium, sodium levels, SGOT, SGPT values, and kidney necrotic cells. Porang flour doses greater than 15,000 mg/kg b.w. caused liver cell damage in white rats and hyperactivity in female rats [108]. There was no toxicity or adverse effects observed in Swiss albino mice when administered with more than 2000 mg/kg of methanolic and aqueous *A. paeoniifolius* tuber extracts (Table 4) [109].

## 7. Concluding Remarks and Future Directions

Research conducted on the genus *Amorphophallus* has demonstrated the therapeutic properties inherent in these particular species. Several plant species within this genus have been employed as traditional medicine for various diseases for a prolonged period. These plants have undergone extensive pharmacological investigations, which have confirmed their medicinal properties. The investigation of phytochemicals within the genus has shown a wide range of compounds that hold promise for developing new drugs. Plants serve as sources of steroids accountable for analgesic, antibacterial, and anticancer properties. Several flavonoids, terpenoids, polysaccharides, and vitamins were extracted from different plant parts, such as tubers, leaves, and seeds. Researchers who conducted in vitro and in vivo experiments have suggested using these plants as antioxidants, as they inhibited free radical generation and showed H_2_O_2_ scavenging activity. The findings suggest that the plant extracts exhibit potential anticancer properties by inducing apoptosis through the downregulation of anti-apoptotic proteins and upregulation of pro-apoptotic proteins. The plants are also possible agents for antibacterial, hypoglycemic, and hepatoprotective activity, owing to the findings that the plant extracts inhibited bacterial growth, inhibited α-glucosidase activity, and reduced hepatic injury, respectively. This is supported by the observed inhibition of bacterial growth, α-glucosidase activity, and reduction of hepatic injury following administration of plant extracts. Significant findings were made concerning these activities.

Moreover, toxicity investigations conducted on biological models have indicated that the use of these plants is potentially safe within physiological systems. The diseases mentioned earlier may benefit from utilizing this genus as a source of novel pharmaceuticals. However, additional research is required to establish the pharmacological advantages of this taxonomic group. It is recommended that additional in vitro and in vivo investigations be undertaken to establish the potential of the genus as a valuable source of medicinal agents. The insufficiency of human clinical data necessitates further investigation. Furthermore, there exist opportunities for conducting toxicity assessments of this genus to assess its safety for human consumption accurately.

## Figures and Tables

**Table 1:** Ethnomedicinal uses of major species of *Amorphophallus*.

Accepted Names	Common Names	Geographical Location	Plant Part Used	Traditional Uses	References
*A. konjac* K. Koch	Devil’s tongue	China, Japan, Southeast Asia	Corm	Detoxification, tumor-suppression, phlegm liquefaction, coughing, asthma, hernia, burns, breast pain, skin, and hematological disorders	[6]
*A. paeoniifolius*	Elephant foot yam, white spot giant arum	India	Corm	Anti-inflammatory, anti-hemorrhoidal, hemostatic, carminative, expectorant, digestive, stomachic, appetizer, anthelmintic, aphrodisiac, liver tonic, emmenagogue, hemorrhoids, rejuvenating and tonic, hemorrhages, coughing, vomiting	[9]
*A. konjac* K. Koch	-	China, Korea, Japan, Indonesia	Tuber	Liver diseases, asthma, piles, abdominal pains, rheumatism, and spleen enlargement	[10]
*A. campanulatus* (Roxb.) Blume	Pungapung, telingo potato, Elephant Yam	Bangladesh	Tuber	Piles, spleen enlargement, asthma, abdominal tumors, boils, abdominal pain, and acute rheumatism	[11]
*A. muelleri*	Porang	Indonesia	Leaves	Astringent, analgesic agent	[12]
*A. bulbifer* (Schott) Blume	Devil’s Tongue	India, Myanmar, Nepal	Rhizomes, petiole, and bulbils	Piles, gonorrhea, hemorrhoids, diarrhea, rheumatic muscular and joint pain	[13]
*A. commutates* var. wayanadensis	Dragon stalk yam	Tropical forest regions of peninsular India	Corms, tubers	Mouth diseases, antidote for snake bites, scabies, antibacterial, hepatoprotective	[14]

**Table 2:** Chemical compounds from the plant part(s) *Amorphophallus* species.

Compound No.	Compounds	Sources	Plant Part(s)	Ref.
Steroids				
**1**	β-sitosterol	*A. campanulatus* (Roxb.) Blume	Whole plant	[18]
		*A. paeoniifolius* (Dennst.) Nicolson	Tuber	[7,19]
**2**	γ-sitosterol	*A. commutatus* var. wayanadensis	Tuber	[14]
**3**	Campesterol	*A. commutatus* var. wayanadensis	Tuber	[14]
**4**	Stigmasterol	*A. commutatus* var. wayanadensis	Tuber	[14]
Flavonoids				
**5**	Vitexin	*A. titanum* (Becc.)	Leaves	[24]
		*A. pusillus* Hett.	Tuber and leaves	[25]
		*A. paeoniifolius* (Dennst.) Nicolson	Inflorescence	[26]
		*A. titanium* (Becc.)	Spathe and spadix appendix	[26]
**6**	Orientin	*A. titanum* (Becc.)	Leaves	[24]
		*A. pusillus* Hett.	Tuber and leaves	[25]
		*A. paeoniifolius* (Dennst.) Nicolson	Inflorescenes	[26]
		*A. titanum* (Becc.)	Spathe and spadix appendix	[26]
**7**	Isovitexin	*A. titanum* (Becc.)	Leaves	[24]
		*A. rivieri* Durieu	Inflorescenes	[26]
		*A. paeoniifolius* (Dennst.) Nicolson	Inflorescenes	[26]
		*A. titanum* (Becc.)	Spathe and spadix appendix	[26]
		*A. rivieri* Durieu	Inflorescenes	[26]
**8**	Isoorientin	*A. titanum* (Becc.)	Leaves	[24]
		*A. titanum* (Becc.)	Spathe and spadix appendix	[26]
		*A. rivieri* Durieu	Inflorescenes	[26]
**9**	Schaftoside	*A. titanum* (Becc.)	Leaves	[24]
		*A. paeoniifolius* (Dennst.) Nicolson	Inflorescenes	[26]
		*A. titanum* (Becc.)	Spathe and spadix appendix	[26]
		*A. pusillus* Hett.	Tuber, leaves	[25]
**10**	Isoschaftoside	*A. titanum* (Becc.)	Leaves	[24]
		*A. titanum* (Becc.)	Spathe	[26]
**11**	Vicenin-2	*A. paeoniifolius* (Dennst.) Nicolson	Inflorescenes	[26]
		*A. titanum* (Becc.)	Leaves	[24]
		*A. titanum* (Becc.)	Spathe	[26]
**12**	Lucenin-2	*A. titanum* (Becc.)	Leaves	[24]
		*A. titanum* (Becc.)	Spathe	[26]
**13**	3,5-Diacetyltambulin	*A. campanulatus* (Roxb.) Blume	Corms	[27]
**14**	Vitexin 2″-O-glucoside	*A. titanum* (Becc.)	Spathe	[26]
**15**	Isovitexin 2″-O-glucoside	*A. titanum* (Becc.)	Spathe	[26]
**16**	Isovitexin 2″-O-rhamnoside	*A. titanum* (Becc.)	Spathe	[26]
**17**	Isoscoparin X″-O-glucoside	*A. titanum* (Becc.)	Spathe	[26]
**18**	Chrysoeriol 7-O-glucoside	*A. titanum* (Becc.)	Inflorescenes	[26]
**19**	Kaempferol 3-O-robinobioside	*A. titanum* (Becc.)	Leaves	[24]
**20**	Quercetin 3-O-robinobioside	*A. titanum* (Becc.)	Leaves	[24]
**21**	Quercetin 3-O-glucoside	*A. paeoniifolius* (Dennst.) Nicolson	Inflorescenes	[26]
		*A. rivieri* Durieu	Inflorescenes	[26]
**22**	Kaempferol 3-O-glucoside	*A. paeoniifolius* (Dennst.) Nicolson	Inflorescenes	[26]
**23**	Fisetin	*A. paeoniifolius* (Dennst.) Nicolson	Tuber	[28]
**24**	Luteolin 7-O-glucoside	*A. titanum* (Becc.)	Leaves	[24]
**25**	Quercetin	*A. bulbifer* (Schott) Blume	Tuber and leaves	[29]
		*A. paeoniifolius* (Dennst.) Nicolson	Corms	[30]
**26**	Cosmosiin	*A. paeoniifolius* (Dennst.) Nicolson	Tuber	[28]
Xanthones				
**27**	Mangiferin	*A. titanum* (Becc.)	Leaves	[24]
**28**	Isomangiferin	*A. titanum* (Becc.)	Leaves	[24]
Phenolic compounds				
**29**	Betulinic acid	*A. paeoniifolius* (Dennst.) Nicolson	Tuber	[19,31,32]
**30**	Tannic acid	*A. konkanensis*	Tuber	[29]
**31**	p-coumaric acid	*A. bulbifer* (Schott) Blume	Tuber, leaves	[33]
		*A. paeoniifolius* (Dennst.) Nicolson	Tuber, leaves	[33]
**32**	Gallic acid	*A. bulbifer* (Schott) Blume	Tuber, leaves	[29]
		*A. paeoniifolius* (Dennst.) Nicolson	Tuber, leaves	[29]
**33**	Resveratrol	*A. paeoniifolius* (Dennst.) Nicolson	Tuber, leaves	[29,33]
**34**	Catechin	*A. bulbifer* (Schott) Blume	Tuber	[29]
**35**	Phenol, 2-methoxy-4-(2-propenyl)-, acetate	*A. sylvaticus* (Roxb.) Kunth	Seeds	[18]
**36**	3,4-dihydroxybenzoic acid	*A. konjac* K. Koch	Roots	[34]
Terpenoids				
**37**	Aromadendrene	*A. impressus* Ittenb.	Inflorescenes	[35]
**38**	Citronellol	*A. pulchellus* Hett. and Schuit.	Inflorescenes	[35]
**39**	Linalool	*A. pulchellus* Hett. and Schuit.	Inflorescenes	[35]
**40**	Nerolidol	*A. pulchellus* Hett. and Schuit.	Inflorescenes	[35]
		*A. hottae* Bogner and Hett.	Inflorescenes	[35]
**41**	2,7-Dimethyl-1,7-octadiene	*A. abyssinicus* (A.Rich.) N.E.Br.	Inflorescenes	[35]
**42**	3,7-Dimethyl 2-octene	*A. abyssinicus* (A.Rich.) N.E.Br.	Inflorescenes	[35]
**43**	Salviasperanol	*A. campanulatus* (Roxb.) Blume	Tuberous roots	[36]
**44**	Amblyone	*A. campanulatus* (Roxb.) Blume	Tuberous root	[37]
**45**	α-Cadinol	*A. sylvaticus* (Roxb.) Kunth	Seeds	[18]
**46**	Cubenol	*A. sylvaticus* (Roxb.) Kunth	Seeds	[18]
**47**	Farnesol	*A. sylvaticus* (Roxb.) Kunth	Seeds	[18]
**48**	α-copaene	*A. sylvaticus* (Roxb.) Kunth	Seeds	[18]
**49**	Caryophyllene	*A. sylvaticus* (Roxb.) Kunth	Seeds	[18]
**50**	Humulene	*A. sylvaticus* (Roxb.) Kunth	Seeds	[18]
**51**	γ-Muurolene	*A. sylvaticus* (Roxb.) Kunth	Seeds	[18]
**52**	Chavibetol	*A. sylvaticus* (Roxb.) Kunth	Seeds	[18]
**53**	α-Cubebene	*A. sylvaticus* (Roxb.) Kunth	Seeds	[18]
**54**	Trans-calamenene	*A. sylvaticus* (Roxb.) Kunth	Seeds	[18]
**55**	Isoaromadendrene epoxide	*A. sylvaticus* (Roxb.) Kunth	Seeds	[18]
**56**	Calarene epoxide	*A. sylvaticus* (Roxb.) Kunth	Seeds	[18]
**57**	Eugenol	*A. sylvaticus* (Roxb.) Kunth	Seeds	[18]
Anthocyanins				
**58**	Cyanidin 3-O-glucoside	*A. titanum* (Becc.)	Spathe	[26]
		*A. rivieri* Durieu	Inflorescenes	[26]
**59**	Cyanidin 3-O-rutinoside	*A. titanum* (Becc.)	Spathe	[26]
		*A. rivieri* Durieu	Inflorescenes	[26]
**60**	Peonidin 3-O-glucoside	*A. titanum* (Becc.)	Spathe	[26]
		*A. rivieri* Durieu	Inflorescenes	[26]
**61**	Peonidin 3-O-rutinoside	*A. titanum* (Becc.)	Spathe	[26]
		*A. rivieri* Durieu	Inflorescenes	[26]
		*A. rivieri* Durieu	Inflorescenes	[26]
**62**	Cyanidin 3-O-rutinoside	*A. pusillus* Hett.	Tuber, leaves	[25]
**63**	Pelargonidin 3-O-glucoside	*A. pusillus* Hett.	Tuber, leaves	[25]
**64**	Peonidin 3-O-rutinoside	*A. pusillus* Hett.	Tuber, leaves	[25]
Vitamins				
**65**	Pyridoxine (Vitamin B6)	*A. paeoniifolius* (Dennst.) Nicolson	Tuber	[28]
**66**	riboflavin	*A. konjac* K. Koch	Corm, dormant stem base	[38,39]
**67**	β-carotene	*A. konjac* K. Koch	Corm, dormant stem base	[38,39]
**68**	Thiamine	*A. konjac* K. Koch	Corm, dormant stem base	[38,39]
**69**	Niacin	*A. konjac* K. Koch	Corm, dormant stem base	[38,39]
Fatty acid and derivatives				
**70**	1-Monopalmitin acid	*A. paeoniifolius* (Dennst.) Nicolson	Tuber	[28]
**71**	Linoleic acid	*A. paeoniifolius* (Dennst.) Nicolson	corms	[40]
		*A. sylvaticus* (Roxb.) Kunth	Seeds	[18]
**72**	α-linolenic acid	*A. paeoniifolius* (Dennst.) Nicolson	Corms	[40]
		*A. sylvaticus* (Roxb.) Kunth	Seeds	[18]
**73**	Lauric acid	*A. commutatus* var. wayanadensis	Tuber	[14]
**74**	Palmitic acid	*A. commutatus* var. wayanadensis	Tuber	[14]
**75**	Stearic acid	*A. commutatus* var. wayanadensis	Tuber	[14]
**76**	Methyl jasmonate	*A. paeoniifolius* (Dennst.) Nicolson	Tuber	[28]
Alkaloid				
**77**	Trigonelline	*A. konjac* K. Koch	Corm, dormant stem base	[38,39]
Hydrocarbon and derivatives				
**78**	n–Heneicosylcyclopentane	*A. lanceolatus* Hett.	Tuber	[41]
**79**	Octadecane	*A. lanceolatus* Hett.	Tuber	[41]
**80**	Heptacosane	*A. lanceolatus* Hett.	Tuber	[41]
**81**	2–Methylfluoranthene	*A. lanceolatus* Hett.	Tuber	[41]
**82**	2(1H) naphthalenone,3,5,6,7,8,8a-hexahydro-4,8a-dimethyl-6-(1-methylethenyl)	*A. lanceolatus* Hett.	Tuber	[41]
**83**	Naphthalene, 1,2,3,5,6,8a-hexahydro-4,7-dimethyl-1-(1-methylethyl)-, (1S-cis)-	*A. sylvaticus* (Roxb.) Kunth	Seeds	[18]
**84**	α-Ketoisocaproic acid	*A. myosuroides*	Inflorescenes	[35]
**85**	Isocaproic acid	*A. angustispathus*, *A. atroviridis*, *A. linearis*, *A. saraburiensis*	Inflorescenes	[35]
**86**	Butanoic acid	*A. taurostigma* Ittenb., Hett. and Bogner	Inflorescenes	[35]
Others				
**87**	Maltitol (Sugar alcohol)	*A. lanceolatus* Hett.	Tuber	[41]
**88**	Lycopersin (polyketide)	*A. lanceolatus* Hett.	Tuber	[41]
**89**	Quinic acid (cyclic polyol)	*A. lanceolatus* Hett.	Tuber	[41]
**90**	Pyrinuron (Nitroaromatic compound)	*A. lanceolatus* Hett.	Tuber	[41]
**91**	2,4,6-Trimethoxyacetophenone (acetophenone)	*A. sylvaticus* (Roxb.) Kunth	Seeds	[18]
**92**	3-Deoxy-d-mannoic lactone (Monosacharide)	*A. paeoniifolius* (Dennst.) Nicolson	Spathe	[40]

**Table 3:** Pharmacological activities of different species of *Amorphophallus*.

Field of Study	Subject	Extracts Used	Dose	Findings	Mechanism of Action	Ref.
Analgesic activity	Swiss albino mice	*A. Paeoniifolius* methanolic extract	250 and 500 mg/kg b.w.	Showed analgesic activity	Cyclooxygenase enzyme is blocked, or opioid receptors in the CNS are stimulated	[20]
		*A. commutatus* methanolic extract	200 and 400 mg/kg	Showed analgesic and antinociceptive activity	Via decreasing TNF-α and COX-2 enzyme activity, prostaglandin synthesis	[54]
		Methanolic extract of *A. campanulatus* tuber	50–500 mg/kg	Showed analgesic activity	-	[11]
CNS effect	Swiss albino mice	*A. paeoniifolius* tuber extract in petroleum ether	100–1000 mg/kg	There was a substantial reduction in sedation activity and locomotor activity	Showed a depressant effect on the CNS by altering the cortex’s function	[56]
		*A. paeoniifolius* petroleum ether extract	100, 300, and 1000 mg/kg	Showed depressive central nervous system impact mediated through the GABAA receptor	Interacting with the subunit to enhance Cl-channel opening mediated by GABA, cell hyperpolarization, and exerting depressive effects on the CNS	[57]
		*A. paeoniifolius* tuber ether extract	100, 300, and 1000 mg/kg	Showed intense CNS depressive action	Reducing locomotor activity	[56]
		*A. paeoniifolius* petroleum ether extract	100, 150, and 200 mg/kg	Showed powerful anxiolytic effects dose-dependently	Because of the presence of steroids, lipids, and fixed oil, both the number of entry and the amount of time spent in open arms increased significantly	[58]
Anti-inflammatory activity	55 male Wister rats	Methanol extract of *A. paeoniifolius*	200 and 400 mg/kg	Exhibited anti-inflammatory activity	Via inhibiting the release of histamine or serotonin	[70]
	Wistar rats and mice	Hydroalcoholic extract of *A. bulbifer*	200 mg/kg	Showed anti-inflammatory properties	Via suppressing prostaglandin synthesis	[13]
Anticonvulsant effect	Isoniazid-induced male albino mice model	Petroleum ether extracts of *A. paeoniifolius*	200, 300, and 400 mg/kg	Induced convulsion onset	By boosting the production and release of GABA, thereby facilitating or inhibiting allosteric receptors	[72]
Antibacterial activity	In vitro	Ethanolic tuber extract of *A. paeoniifolius*	15 μL, 20 μL, and 25 μL	Inhibited growth of bacteria	-	[73]
	4 Gram (+)ve bacteria, 6 Gram(−)ve (in vitro)	Amblyone separated from *A. campanulatus*	80–160 μg/disc	Amblyone was most active against *Bacillus megaterium* and least active against *Pseudomonas aeruginosa.*	-	[37]
	Bacterial cultures (in vitro)	Extract of *A. paeoniifolius*	20 µL extracts	*B. subtilis* MTCC 121 and *S. aureus* MTCC 737 exhibited the most activity with ethyl acetate extract	Because of the extract’s glycosides, phenols, polysterols, tannins, flavonoids, terpenoids, gum, steroids, and mucilage	[22]
	*S. aureus*, *B. subtillis*, *P. aeruginosa*, *S. typhimurium*, *E. coli*, *C. freundii*	*A. paeoniifolius* tuber extract	25 µL of tuber-mediated synthesized nanoparticles	Showed significant antibacterial activity	-	[74]
Hepatoprotective activity	Albino Wistar rats	*A. campanulatus* tuber methanolic extract	250–500 mg/kg	Increasing levels of SOD, CAT, and GPx observed hepatoprotective and antioxidant properties	Reducing hepatic injury biomarkers such as SGPT, SGOT, ALP, BRN, and total protein shows hepatoprotective activity	[61]
	Wistar rats	*A. campanulatus* extract	250–500 mg/kg	Protected rat hepatic tissue against ethanol-induced oxidative damage, most likely by acting as an antioxidant	There is a decrease in serum marker enzymes, such as aspartate transaminase, alanine transaminase, and alkaline phosphatase. The levels of SOD, CAT, and GPx all increased dramatically	[21]
	Male albino Wistar rats	Aqueous and methanol extracts of *A. paeoniifolius* tubers	300 mg/kg	Demonstrated hepatoprotective activity in the presence of paracetamol-induced liver damage	Flavonoids and steroids may be accountable for the hepatoprotective effect	[21]
	Male albino Wistar rats	Aqueous and ethanol extracts of *A. campanulatus* (Roxb.)	500 mg/kg was given orally once a day	Demonstrated a robust hepatoprotective effect in tetrachloride-induced liver damage	Because the extracts contain flavonoids, which have the potential to scavenge free radicals	[62]
	Wistar rats	*A. campanulatus* Roxb. ethanol extract	250–500 mg/kg	Inhibited ethanol-induced oxidative damage in rat hepatic tissue, most likely through antioxidant activity	Via reducing oxidative damage to hepatocytes by ROS scavenging activity, it also restores SOD and CAT activity in liver tissue, normalizes the levels of liver marker enzymes, reduces GSH depletion and TBARS formation, and preserves SOD and CAT activity	[63]
	Swiss albino mice	*A. commutatus* methanol extract	100–400 mg/kg	Revealed a vigorous antioxidant activity, hepatoprotective effect, and lowered lipid peroxidation	Antioxidant enzyme activity increases, lipid peroxidation is inhibited, and hepatic marker levels are decreased	[64]
Antioxidant	Wistar rats (in vitro)	Ethanol extract of *A. paeoniifolius*	1–50 µg/mL	It has a significant inhibitory effect on lipid peroxidation	Inhibiting free radical generation by oxidation and H_2_O_2_ scavenging activity. Gallic acid, resveratrol, and quercetin, among the phenolic components included in the extract, may contribute to the scavenging activity	[33]
	Wistar mice	Methanol extracts of the *A. campanulatus*	125–250 mg/kg	Showed curative effect against TAA-induced OS in rats	Reducing serum ALT, LDH, AST, ALP, and tissue malondialdehyde levels	[77]
	Rats	Hexane and methanol extract of *A. campanulatus*	125 and 250 mg/kg	Exhibit radical scavenging and antioxidant action	ACME dramatically reduced serum AST, ALT, ALP, LDH, and tissue malondialdehyde levels. GSH, GST, GR, GPx, and CAT levels were significantly elevated in the liver and kidneys	[78]
Immunomodulatory Activity	Rats	*A. campanulatus* methanol extract	250 and 500 mg/kg	Suppressed the immune system dose-dependently	Reducing charcoal clearance, DTH response, and spleen index	[104]
Antihelmintic activity	*Pheretima posthuma*, an adult Indian earthworm	Methanolic extracts of *A. paeoniifolius*	25, 50, and 100 mg/mL	Showed antihelmintic efficacy against *Pheretima posthuma*	-	[105]
Preventing gastrointestinal disturbances (Anti-diarrheal activity and Laxative effect)	Wistar mice	Methanolic extracts of *A. paeoniifolius*	250 and 500 mg/kg	It has gastrokinetic activity and has moderately increased the number of stools, feces volume, fecal matter water content, gastric emptying, and intestinal transportation	The spasmogenic and stimulatory effect on the synthesis of prostaglandins was detected due to betulinic acid and glucomannan in the extract	[31]
		Methanolic extracts of *A. paeoniifolius*	125, 250, and 500 mg/kg	Treatment with tuber extracts, glucomannan, and betulinic acid in constipated rats resulted in a considerable improvement in fecal parameters and intestinal transit	By partial agonistic action in 5HT receptors	[7]
	Male albino rats	Konjac flour	300 and 600 mg/kg	Effective in influencing increased water content, fecal pellet weight, and intestinal motility, demonstrating laxative activity	Because of its ability to absorb and retain water in the gastrointestinal tract	[82]
Anticancer and antitumor effect	HT-29 cell line (in vitro)	*A. commutatus* methanolic extract	50 μg/mL	MEAC increased antioxidant activity as well as cytotoxicity against HT-29 cells.	Initiating the caspase-3-dependent apoptotic pathway, inducing a G1/S cell cycle arrest, and subsequently downregulating the COX-2 pathway	[84]
	Tumor-bearing mice model	*A. commutatus* methanolic extract	400 mg/kg	Anticancer activity	Increasing antioxidant enzymes, bringing the hematological profile back to normal. The COX-2 and caspase-dependent pathways mediate the anticancer action of β-sitosterol extracted from ACW	[84]
	MCF-7 and MDA-MB-231 cell lines	*A. paeoniifolius* (Dennst.) extract	10 and 20 mg/mL	Significant cytotoxic activity was shown to be dose and time-dependent	Apoptosis was increased in these cells, as evidenced by a decrease in antiapoptotic Bcl-2 and an increase in pro-apoptotic Bax, Caspase-7 expression, PARP breakdown, and decreased motility in both cell lines	[28]
	SGC-7901, HEK293, and AGS cell lines	The extract of tuber of *A. konjac* (TuAK)	25–100 µg/mL	Significantly slowed the growth of cultured gastric cancer cell lines	Survivin and Bcl-2 expression was reduced, while Bax and caspase-9 expression were increased in the apoptosis-associated proteins	[87]
	Human colon carcinoma cell line HCT-15 (in vitro)	*A. campanulatus* (*Roxb.*) Bl. tuber extracts	10 mg	ACME subfractions inhibited HCT-15 cell proliferation and caused apoptosis in a dose-dependent manner	Through two fundamental mechanisms: change of redox state and interference with essential cellular functions	[85]
	Male Wistar rats	Methanol extracts of the *A. campanulatus*	125 and 250 mg/kg	Significantly cut down on the rise in hepatic nodules, nodule multiplicity, and serum biochemical markers caused by NDEA, as well as improved hepatocellular architecture	Through the restoration of antioxidant enzymes SGPT, SGOT, ALP, BRN, and total protein, as well as peroxide radical scavenging activity	[88]
	Male Wistar rats	Methanol extracts of the *A. campanulatus*	250 mg/kg body weight orally	Has an enormous effect on chemoprotection	Significant lipid peroxidation in the intestine and colon and reduced antioxidants such as CAT, GPx, GR, GST, and GSH	[86]
Hypoglycemic activity	Wistar rats	*A. konjac* extract	102 mg/Kg o	Helps improve glucose metabolism by blocking the enzyme -glucosidase, which improves glucose metabolism by lowering blood sugar	By suppression of lipid peroxidation; inhibition of α-amylase and α-glucosidase activities; improvement of GPx and CAT activities; and reduction of malondialdehyde, lactate dehydrogenase, and reactive oxygen species aggregation	[93]
	Swiss albino mice	Methanol extract of corms of *A. campanulatus*,	50, 100, 200, and 400 mg per kg body weight	Blood glucose levels were significantly lowered	Because of α-glucosidase and α-amylase inhibitory action of betulinic acid, lupeol, stigmasterol, and β-sitosterol	[94]
	Wistar rat	Glucomannan from *Amorphophallus*	60 mg/kg/body weigh/day	*A. variabilis*’ diet may have lowered blood cholesterol levels	-	[95]
	Albino rats	*Vigna radiata* (L.) R. Wilczek and *A. paeoniifolius* (Dennst.) combination	0.054 g and 0.216 g per 100 g body weight	Lowered cholesterol, triglycerides, low-density lipoprotein, and levels while increasing HDL	Lowering the levels of the hormone leptin with competitive inhibition of HMG-CoA reductase and reduction of LDL production	[96]
	30 Sprague Dawley rats	Porang Glucomannan Supplementation	25–100 mg/200 g BW	Enhanced lipid profile in rats induced with metabolic syndrome	Lowering total cholesterol (TC), triglycerides (TG), and LDL, while increasing HDL	[97]
Neuroprotective effect	Rat	*A. campanulatus* petroleum ether extract	100, 200, and 500 mg/kg, p.o. for 14 days	Exhibited neuroprotective effect	Lowering oxidative stress (SOD, CAT, and LPO), amyloid peptide (A), and acetylcholinesterase (AchE) levels in brain tissue	[59]
Preventing renal damage	Male albino rats of the Wistar strain	Ethanolic extract of *A. campanulatus* a	250 and 500 mg/kg	The antioxidative and antiapoptotic properties of the extract were found to be effective in reducing ACE-induced nephrotoxicity	Serum urea, creatinine, pro-inflammatory cytokines, tissue TBARS, and GSH metabolizing enzyme activity were dramatically increased, but cytosolic and mitochondrial SOD, CAT, and reduced GSH levels were significantly lowered	[79]
Skin protection	51 healthy humans	*A. konjac* extract capsules (5 mg glycosylceramides)	100 mg/day	Improved skin metrics such as dryness, hyperpigmentation, irritation, and oiliness	Sphingolipids consumed are transformed into sphingolipid metabolites, which are absorbed by the colon and circulated in the blood	[106]
	Male NC/Nga mice, a murine model of human AD (in vitro)	5% (*w*/*w*) of konjac GM powders	300 µm, 100 µm, 160 µm	Atopic illnesses such as dermatitis, asthma, allergic rhinitis, skin inflammation, and hyper-IgE production were inhibited	IFN-α, a positive regulatory cytokine of atopic skin inflammation, is suppressed through systemic downregulation	[71]
Antiobesity Activity	Wistar rat	*A. konjac* refined powder	-	Its antioxidation is considerably enhanced, and serum lipid levels are reduced; however, the antiobesity effect is limited	In rats, decreasing MDA levels and significantly increasing SOD and GSH-Px activities, as well as lowering TC, LDL, TG levels, and rising HDL levels, reduced MDA levels and significantly increased SOD and GSH-Px activities	[100]
	Sprague Dawley rats	Konjac flour, a powder from the tuber of the *Amorphophallus*	100 g	Decreased body weight, glucose, triglyceride, and HDL levels in their blood	Slowing stomach emptying, attaching to bile acids in the gut, and transporting them out of the body in the feces	[38]
	96 SD white rat	*A. konjac*	0.2–0.8 (g kg^−1^)	Reduces rat body weight, triglyceride, glucose, cholesterol, and HDL levels in the blood	Delay stomach emptying and more gradual dietary sugar absorption	[101]
	Individuals who are overweight or obese	Glucomannan	2–4 g per day	If used in conjunction with a regular caloric or hypocaloric diet, it helps people lose weight	Due to increased viscosity of gastrointestinal content, delayed stomach emptying, and decreasing colon transit time	[102]
	58 obese subject	Glucomannan fibers, abundant in *A. konjac*	Konjac (1.5 g) daily for 12-week period	Cured obesity-related dyslipidemia because of the cholesterol-lowering action of *Garcinia cambogia* and *A. konjac* combined with their apparent low toxicity	Promoting weight loss, potentially reducing fatty acid biosynthesis, increasing water-absorbing ability, and decreasing stomach emptying speed, altering the kinetics of duodenal fat absorption	[103]

Abbreviations: Bilirubin = BRN, Catalase = CAT, Delayed-type hypersensitivity = DTH, Gutathione peroxidase = GPx, Glutathione = GSH, Glutathione-S-transferase = GST, Glutathione reductase = GR, Methanolic extract of *A. campanulatus* = MEAC, Methanolic extracts of *A. paeoniifolius* = APME, Methanol extracts of the *A. campanulatus* = ACME, Oxidative stresses = OS, Petroleum ether extracts = PEEs, Serum oxaloacetate transaminase = SGOT, Superoxide dismutase = SOD, Serum glutamate pyruvate transaminase = SGPT, Terpene synthase = TPS, Thioacetamide = TAA, Tuber extracts of *A. konjac* = TuAK, Thiobarbituric acid reactive substances = TBARS, *A. paeoniifolius* = *Amorphophallus paeoniifolius*, *A. konjac* K. Koch—*Amorphophallus konjac*, *A. campanulatus = Amorphophallus campanulatus*, *A. rivieri* = *Amorphophallus rivieri*, *A. konkanensis* = *Amorphophallus konkanensis*, *A. sylvaticus* = *Amorphophallus sylvaticus*, *A. sylvaticus* = *Amorphophallus sylvaticus*, *A. titanium* = *Amorphophallus titanium*, Aqueous extracts of *A. paeoniifolius* = APAE, Alkaline phosphatase = ALP, *A. commutatus* var. *wayanadensis* = ACW.

**Table 4:** Safety and toxicity studies of different *Amorphophallus* species.

Species	Dose of Toxicity	Study Model	Outcomes	References
APME and APAE tuber	>2000 mg/kg	Wistar mice	The LD50 was above 2500 mg/kg	[31]
*A. paeoniifolius*	>1.5 g/kg	Swiss albino mice	Non-toxic up to 1.5 g/kg	[58]
Porang (*A. oncophyllus*)	>5000 mg/kg BW	Rats	Not detrimental until the maximum dose of 5000 mg/kg b.w.	[107]
Porang (*A. muelleri Blume*)	>15,000 mg/kg BW	White rats (*Rattus norvegicus*)	Greater than 15,000 mg/kg BW porang flour dosage caused liver cell damage in white rats, and hyperactivity among the female rats was also observed	[108]
APME and APAE tuber	>2000 mg/kg	Swiss albino mice	No toxicological property was reported at the doses tested	[109]

## Data Availability

Available data are presented in the manuscript.

## Abbreviations

APAE	Aqueous extracts of *A. paeoniifolius*
ALP	Alkaline phosphatase
BRN	Bilirubin
CAT	Catalase
DTH	Delayed-type hypersensitivity
GPx	Gutathione peroxidase
GSH	Glutathione
GST	Glutathione-S-transferase
GR	Glutathione reductase
MEAC	Methanolic extract of *A. campanulatus*
APME	Methanolic extracts of *A. paeoniifolius*
ACME	Methanol extracts of the *A. campanulatus*
OS	Oxidative stresses
PEEs	Petroleum ether extracts
SGOT	Serum oxaloacetate transaminase
SOD	Superoxide dismutase
SGPT	Serum glutamate pyruvate transaminase
TPS	Terpene synthase
TAA	Thioacetamide
TuAK	Tuber extracts of *A. konjac*
TBARS	Thiobarbituric acid reactive substances
